# Supplementary material for: Common mechanism of transcription termination at coding and noncoding RNA genes in fission yeast
Source: Nat Commun. 2018 Oct 19;9:4364. doi: 10.1038/s41467-018-06546-x (PMC6195540; doi:10.1038/s41467-018-06546-x)
Supplement: Supplementary file 1 — Supplementary Information [file 41467_2018_6546_MOESM1_ESM.pdf]

SUPPLEMENTARY INFORMATION

Common mechanism of transcription termination at coding and noncoding RNA genes in fission yeast. Marc Larochelle et al.

Includes:

1. **SUPPLEMENTARY FIGURES**
2. **SUPPLEMENTARY TABLES**
3. **SUPPLEMENTARY UNCROPPED IMAGES**
4. **SUPPLEMENTARY REFERENCES**

SUPPLEMENTARY FIGURES

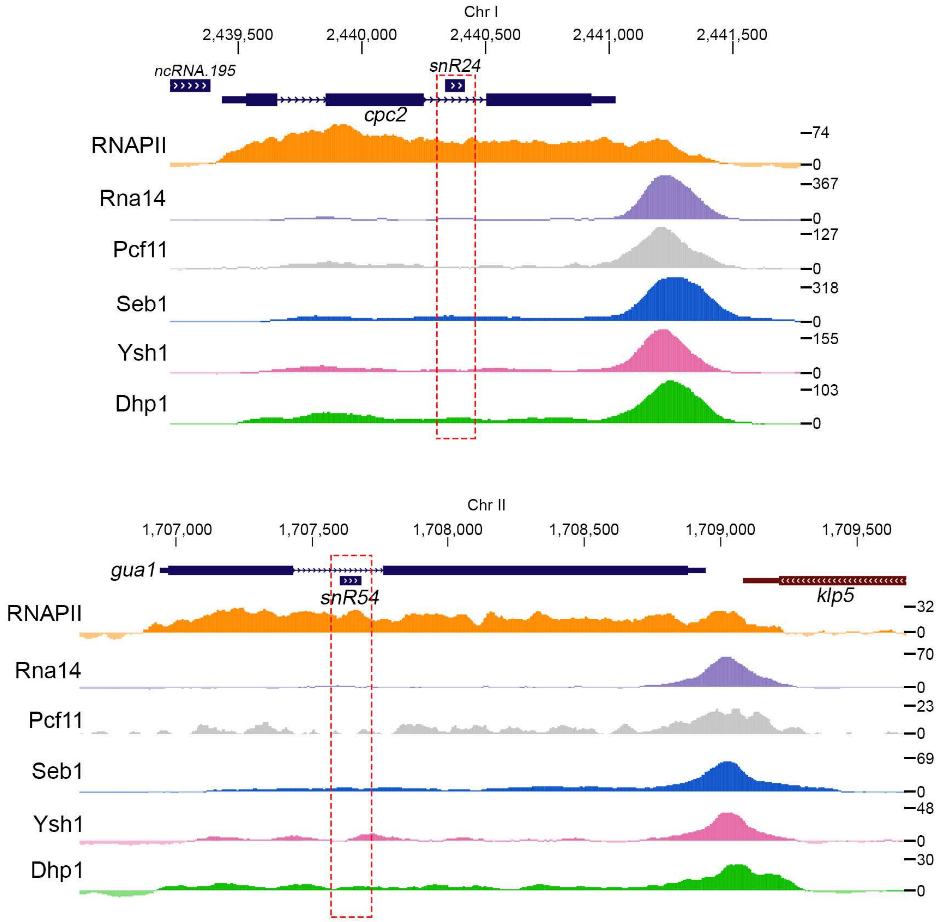

Supplementary Fig. 1. *S. pombe* mRNA 3' end processing and transcription termination factors are not recruited to intronic snoRNA genes. Normalized ChIP-seq signal of total RNAPII as well as the indicated mRNA 3' end processing and transcription termination factors across the *cpc2* (top) and *gua1* (bottom) mRNA genes. Dashed-line red rectangles show the absence of Rna14, Pcf11, Seb1, Ysh1, and Dhp1 enrichment at intronic *snR24* (top) and *snR54* (bottom) snoRNA genes.

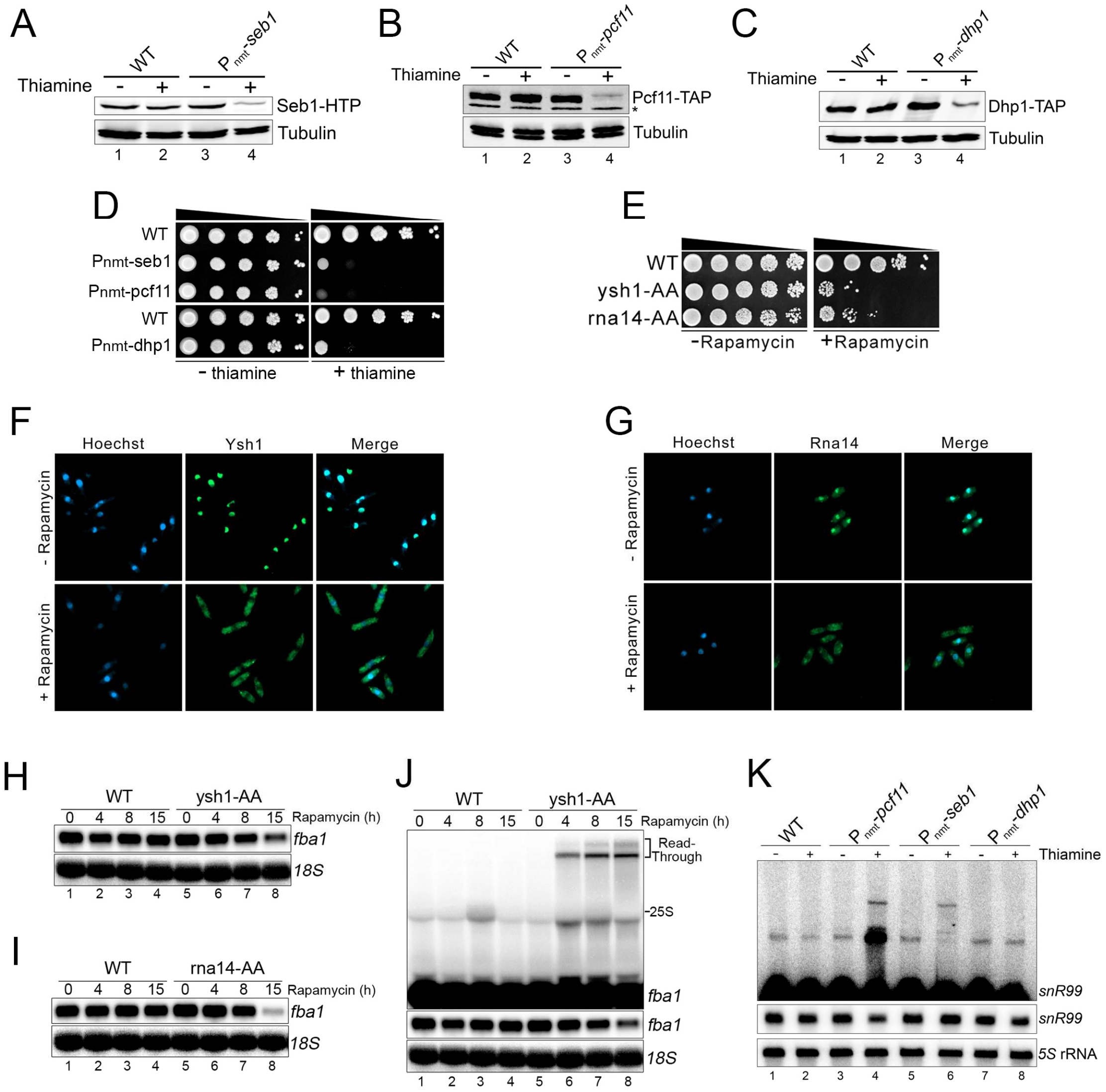

Supplementary Fig. 2. Establishment of conditional fission yeast strains for Seb1, Pcf11, Dhp1, Rna14, and Ysh1. (A-C) Western blot analysis of chromosomally-tagged Seb1 (A), Pcf11 (B), and Dhp1 (C) expressed from their endogenous (lanes 1-2) or *nmt* promoter (lanes 3-4) in the absence (lanes 1 and 3) and presence (lanes 2 and 4) of thiamine to inactivate the *nmt* promoter. The asterisk corresponds to a non-specific protein. (D) Ten-fold serial dilutions of wild-type (WT), P_{nmt}-*seb1*, P_{nmt}-*pcf11*, and P_{nmt}-*dhp1* cells were spotted on thiamine-free (left) or thiamine-containing (right) minimal media. (E) Ten-fold serial dilutions of wild-type (WT), *ysh1* anchor-away *(ysh1-AA),* and *rna14-AA* cells were spotted on rapamycin-free (left) or rapamycin-containing (right) minimal media. (F-G). Representative pictures of Ysh1-FRB-GFP (F) and Rna14-FRB-GFP (G) re-localization from the nucleus to the cytoplasm 3h after rapamycin treatment (bottom panels), whereas nuclear localization was observed in the absence of Rapamycin (top panels). (H-I) Rapamycin-dependent inhibition of mRNA synthesis in *ysh1-AA* (H) and *rna14-AA* (I) strains. Northern blot analysis of total RNA prepared from WT and the indicated anchor-away mutants at 0h, 4h, 8h, and 15h after treatment with rapamycin. The blot was hybridized with an antisense RNA probe complementary to the *fba1* mRNA. (J) Rapamycin-dependent accumulation of *fba1* read-through transcripts in the *ysh1-AA* strain. Note the accumulation of *fba1* read-through transcripts 4h after the addition of rapamycin in the *ysh1-AA* strain (lanes 5-8), but not in the wild-type (WT) strain (lanes 1-4). (H-J) The 18S rRNA was used as a loading control. (K) RNA prepared from the indicated strains that were grown without thiamine (-) or in thiamine-supplemented medium (+) for 15h to deplete Pcf11, Seb1, and Dhp1. Total RNA was treated with RNase H in the presence of DNA oligonucleotides complementary to *snR99* in the presence of oligo d(T). The top panel represents a longer exposure of the middle panel to see 3'-extended snoRNA precursors. The 5S rRNA was used as a loading control.

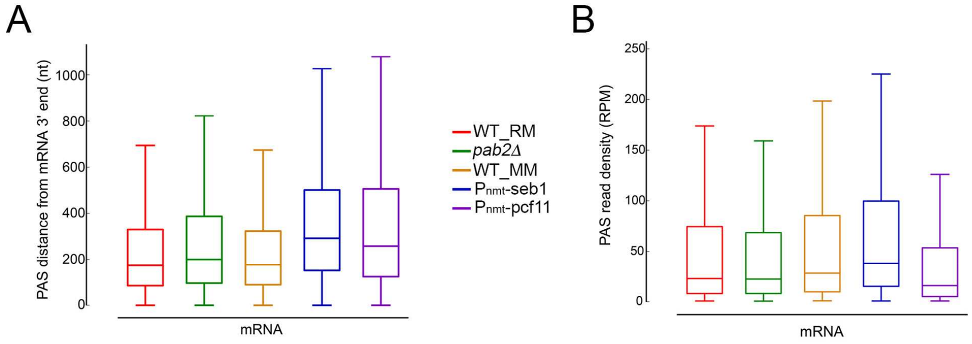

C

End of *snR99* gene *rps2* terminator

5′GGATTGTCTCCCAGACATTTACTAGTGACGCTGAAAAGCAAAGTACTTTTTCTTTTCAAT

TGTACATTGTTGTAAAGCCTTCCAGCTTTAATAAAATAAAAAAGTTAACT**C**AAAG**CT**AGTTT

ATCTTTTGTTCAGTAACGTGTATATTGGTATCAGTCAATTTTTCATAGTCACTGTAAGTAAG

TTGCACAACAT 3′

D

End of *snR99* gene *trx1* terminator

5′GGATTGTCTCCCAGACATTTACTAGTTTTACGCAATCTGATAGTTAGTTGTTATGAAC

TTGTAATACAAAATATTTTTATTTAACAAAAA**T**AAATGTTTTGCTTTT**CTT**T**G**T**GT**A**T**GC

ACCGTTGTAATTTATTGGGAAAAGTATTTTTGTCCTAGCATCTTTTCTACTGAAGTTGCA

GAACGGCGATTTGCGT 3′

Supplementary Fig. 3. Effects of Pab2, Seb1, and Pcf11 deficiencies on mRNA polyadenylation and cleavage site analysis of *snR99-rps2* and *snR99-trx1* chimeric constructs. (A-B) Box-plots showing the distribution of the distance calculated between the strongest poly(A) site (PAS) and the mRNA stop codon as determined by 3'READS (A) and the sum of the read density for all of the poly(A) sites associated to a mRNA in each condition (B). Center lines correspond to the median. (C-D) Results of 3' RACE analyses using strains that expressed the *snR99-rps2* (C) and *snR99-trx1* (D) chimeric constructs. Nucleotides in red correspond to the polyadenylation sites used by the endogenous *rps2* and *trx1* genes as determined by genome-wide approaches(1,2). Arrows indicate the position of the polyadenylation site used in the *snR99-rps2* (C, n=3 clones) and *snR99-trx1* (D, n=6 clones) chimeric constructs as determiend by 3' RACE assays.

ChIP target	Pearson Correlation Coefficients		
Ser5-P B1	1	0.961	
Ser5-P B2	0.961	1	
Ser7-P B1	1	0.9698	
Ser7-P B2	0.9698	1	
Ser2-P B1	1	0.9613	
Ser2-P B2	0.9613	1	
Tyr1-P B1	1	0.9572	
Tyr1-P B2	0.9572	1	
Rpb1 B1	1	0.941	0.9276
Rpb1 B2	0.941	1	0.9614
Rpb1 B3	0.9276	0.9614	1
Rpb3 B1	1	0.9329	
Rpb3 B3	0.9329	1	

Supplementary Fig. 4. Genowe-wide correlation between ChIP-seq experiments. Genome-wide Pearson correlation coefficients between independent ChIP-seq experiments using antibodies targeting the indicated proteins or CTD modifications (B1, batch 1; B2, batch 2; B3, batch 3).

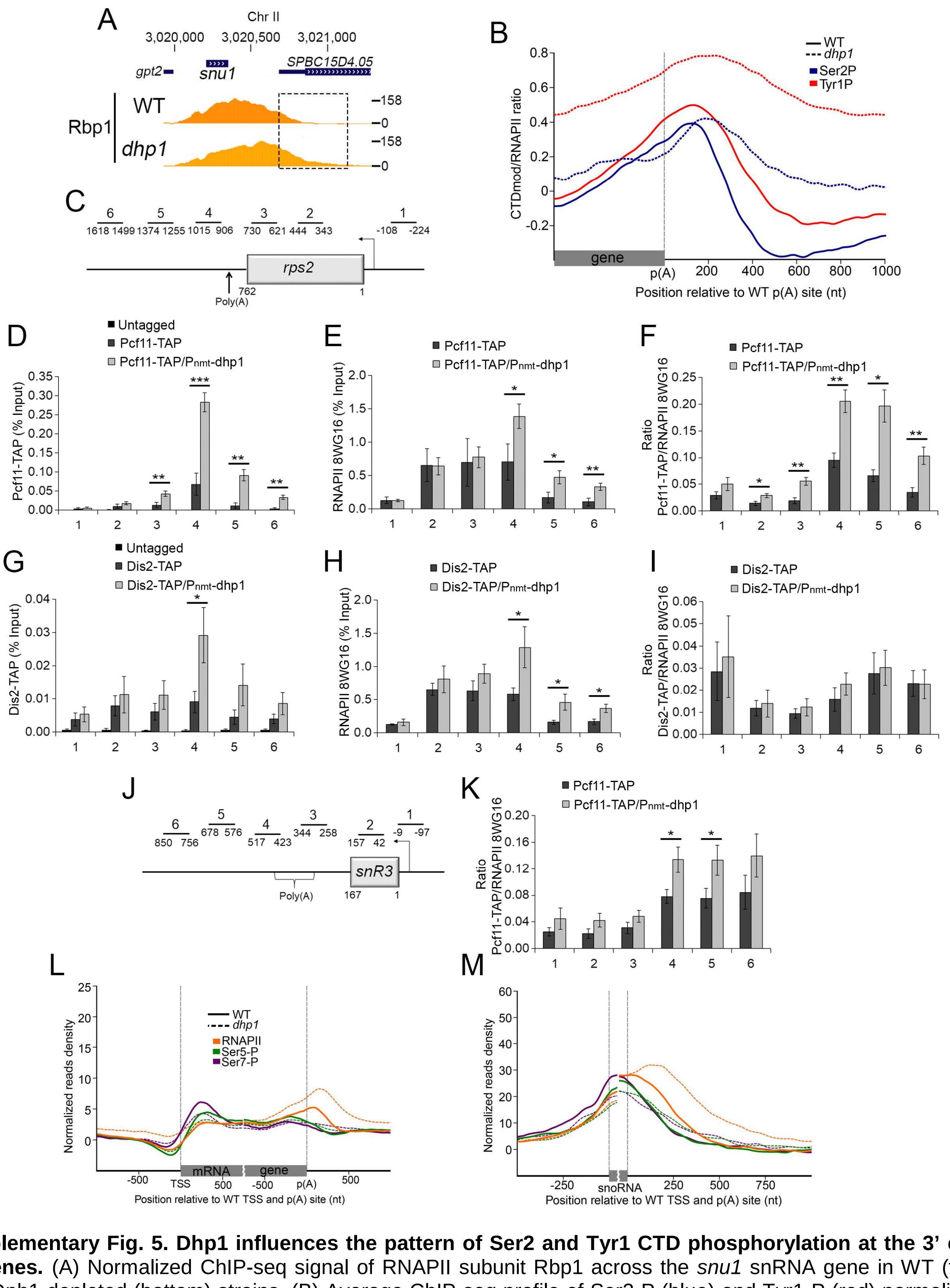

Supplementary Fig. 5. Dhp1 influences the pattern of Ser2 and Tyr1 CTD phosphorylation at the 3' end of genes. (A) Normalized ChIP-seq signal of RNAPII subunit Rbp1 across the *snu1* snRNA gene in WT (top) and Dph1-depleted (bottom) strains. (B) Average ChIP-seq profile of Ser2-P (blue) and Tyr1-P (red) normalized by the RNAPII signal in a WT (solid lines) and Dhp1-depleted strain (dotted lines) centered on the polyA sites (p(A)) of 4,755 mRNA and 24 snoRNA genes with a PAS in minimal medium. (C) Bars above the *rps2 gene* show the positions of PCR products used for ChIP-qPCR analyses in panels D-I. (D) ChIP assays using a TAP-

tagged version of Pcf11 or an untagged control strain at the *rps2* gene in wild-type and P_{nmt}-*dhp1* strains in the presence of thiamine to deplete Dhp1. Input and copurified DNA were quantified by qPCR using primers shown in panel C. (E) ChIP assays performed using an RNAPII-specific antibody at the *rps2* gene in wild-type and P_{nmt}-*dhp1* strains in the presence of thiamine to deplete Dhp1. (F) Density of Pcf11 relative to RNAPII at the *rps2* gene in wild-type and P_{nmt}-*dhp1* strains in the presence of thiamine. Data and error bars represent the average and standard deviation from three biological replicates. (G-I) Same as for D-F but using a TAP-tagged version of Dis2 instead of Pcf11. (J) Bars above the *snR3 snoRNA gene* show the positions of PCR products used for ChIP-qPCR analyses in panel K. (K) Density of Pcf11 relative to RNAPII at the *snR3* gene in wild-type and P_{nmt}-*dhp1* strains in the presence of thiamine. Data and error bars represent the average and standard deviation from three biological replicates. *: $p < 0.05$; **: $p < 0.01$; ***: $p < 0.001$ (Student's t-test). (L-M) Average ChIP-seq profile of total RNAPII (Rpb1, orange), Ser5-P (green), Ser7-P (purple) in WT (solid lines) and Dhp1-depleted (dotted lines) strain across 4,755 mRNA (L) and across 24 snoRNA (M) with a PAS identified in minimal medium.

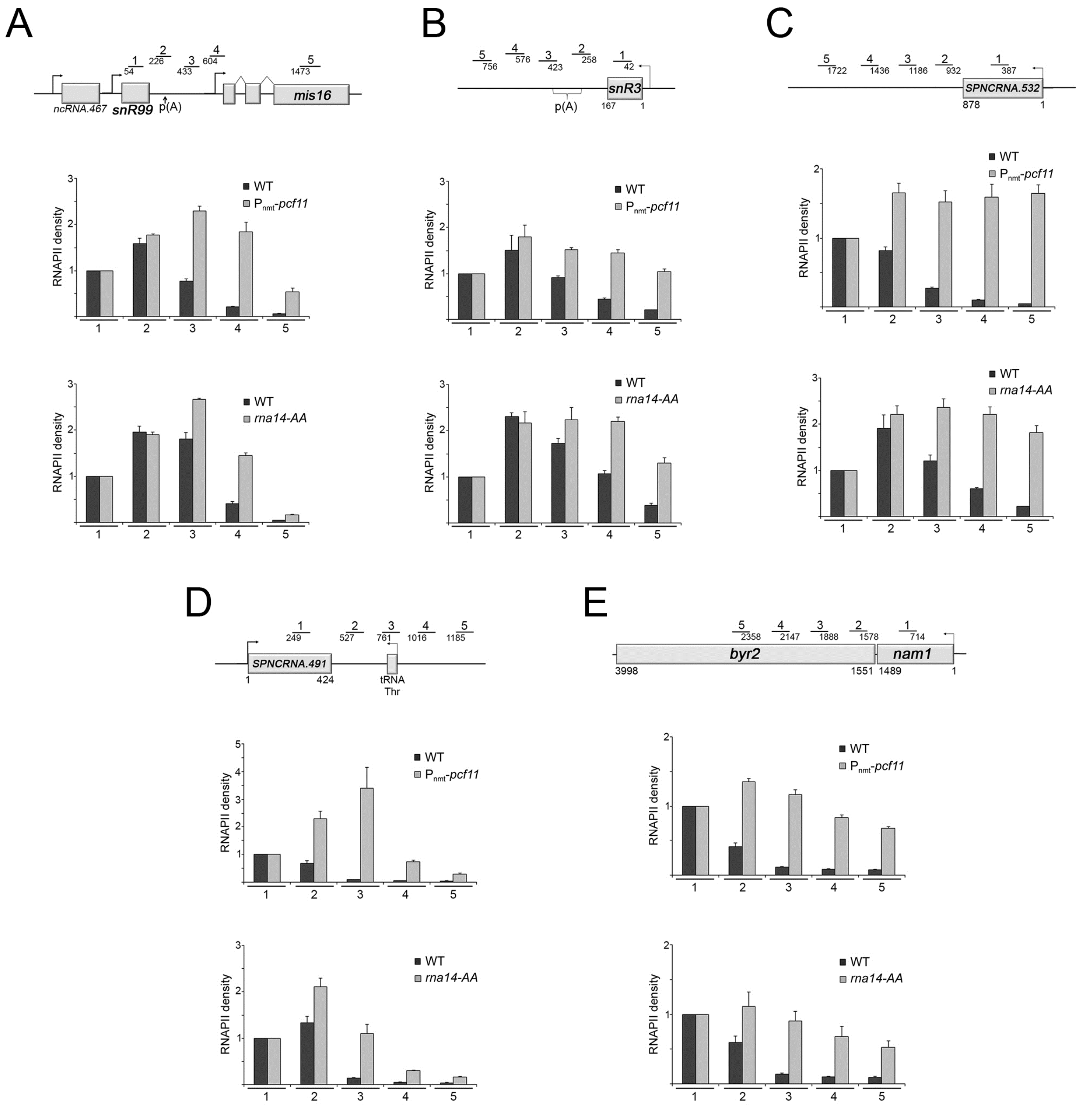

Supplementary Fig. 6. Read-through transcription at ncRNA genes in cells deficient for Pcf11 and Rna14. (A-C) RNAPII ChIP-qPCR analysis on the *snR99* (A) and *snR3* (B) snoRNA genes as well as the *SPNCRNA.532* (C), *SPNCRNA.491* (D), and *SPNCRNA.1459/nam1* (E) lncRNAs using extracts prepared from either wild-type (WT) or *pcf11* (*Pnmt-pcf11*; top graph) and *rna14* (*rna14-AA;* bottom graph) mutant strains. Bars above the *genes* show the positions of PCR products used for ChIP-qPCR analyses. Cells were grown in the presence of thiamine for 15h for the analysis in the *Pnmt-pcf11* strain and in rapamycin for 4h for the *rna14-AA* strain. ChIP signals (percent of input) were normalized to region 1. Error bars indicate SD. *n* = 3 biological replicates from independent cultures.

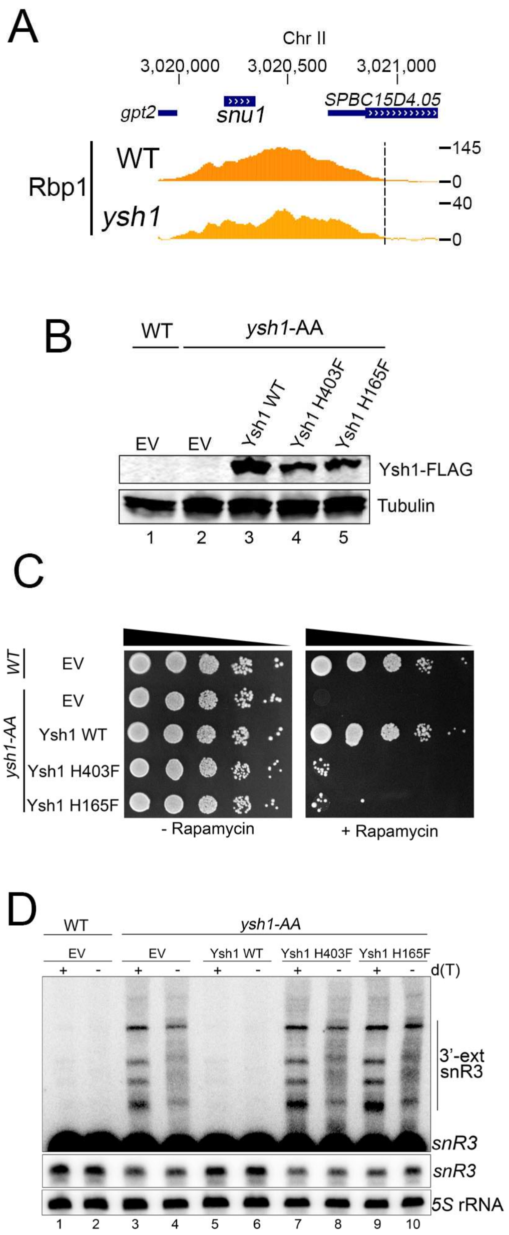

Supplementary Fig. 7. The endonucleolytic activity of Ysh1 is required for snoRNA synthesis. (A) Normalized ChIP-seq signal of RNAPII subunit Rbp1 across the *snu1* snRNA gene in WT (top) and Ysh1-depleted (bottom) strains. (B) Western blot analysis of extracts prepared from WT cells (lane 1) as well as from *ysh1-AA* cells (lanes 2-5) in which an empty vector (EV) or constructs that express FLAG-tagged versions of WT Ysh1 (lane 3), H403F (lane 4) and H165F (lane 5) mutant versions of Ysh1. Cells were grown in the presence of rapamycin to deplete endogenous Ysh1. Tubulin was used as a loading control. (C) Ten-fold serial dilutions of wild-type and *ysh1-AA* cells that were transformed with an empty vector (EV) or constructs that express wild-type (WT) and mutant versions of Ysh1 were spotted on rapamycin-free (left) or rapamycin-containing (right) media. (D) Total RNA prepared from wild-type (lanes 1-2) and *ysh1-AA* (lanes 3-10) strains was treated with RNase H in the presence of a DNA oligonucleotide complementary to H/ACA class snoRNA *snR3*. RNase H reactions were performed in the presence (+) or absence (−) of oligo(dT). 5S rRNA was used as a loading control. Expression of catalytically inactive versions of Ysh1 resulted in reduced levels of mature *snR3* and the accumulation of 3-extended polyadenylated *snR3* precursors (lanes 7-10) similar to *ysh1-AA* cells transformed with the empty vector (EV) control (lanes 3-4).

A

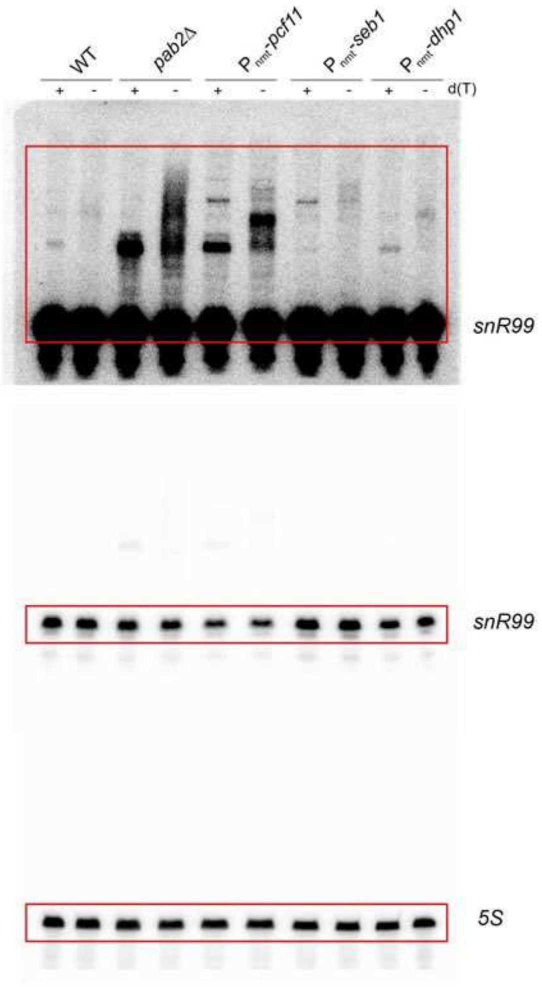

Uncropped images
of Fig. 2C

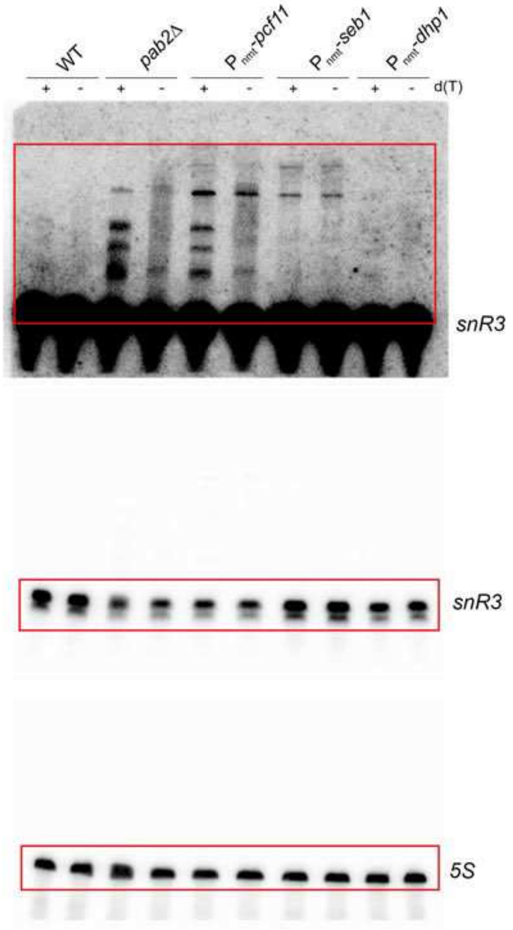

Uncropped images
of Fig. 2B

B

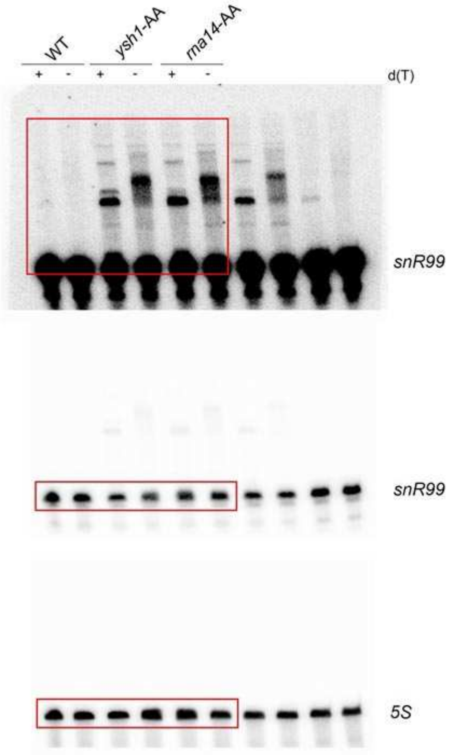

Uncropped images of
Fig. 2E

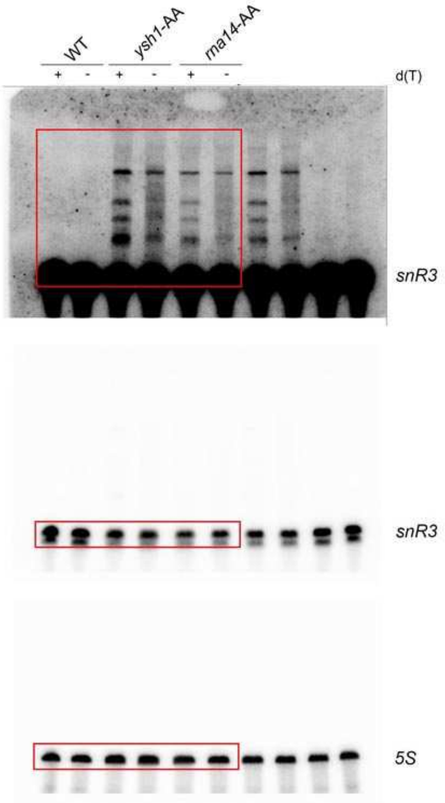

Uncropped images of
Fig. 2D

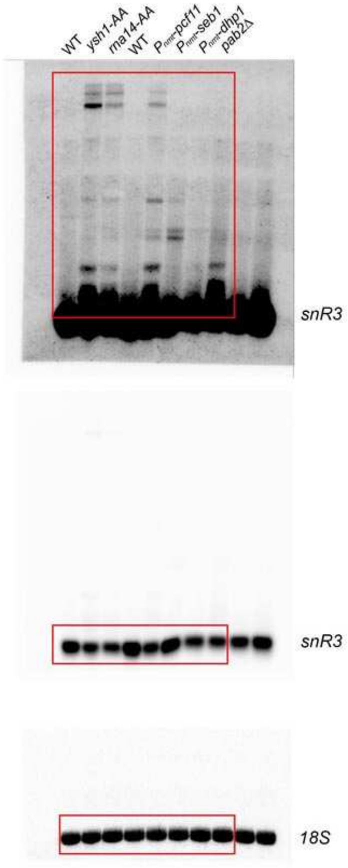

Uncropped images of
Fig. 2F

C

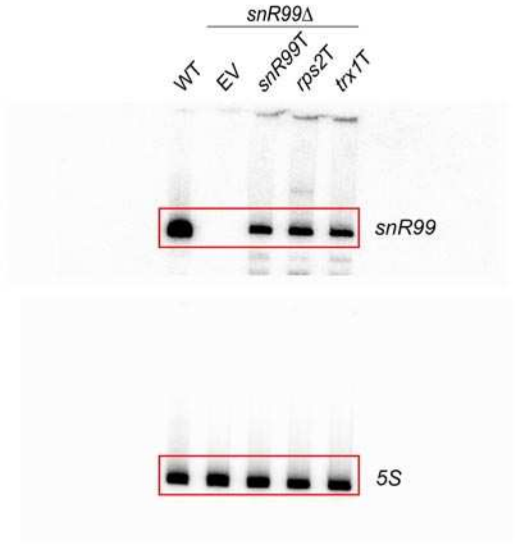

Uncropped images
of Fig. 3F

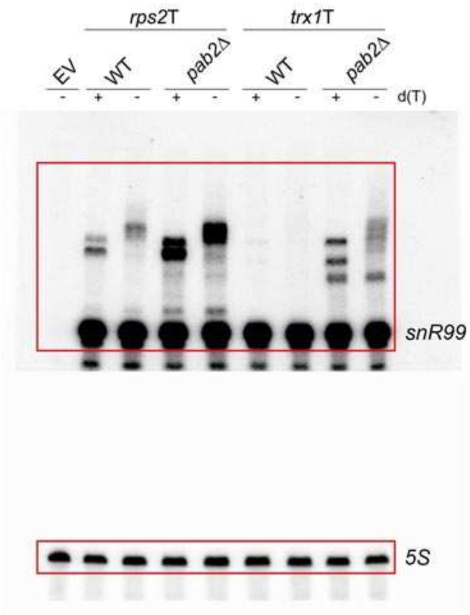

Uncropped images
of Fig. 3G

D

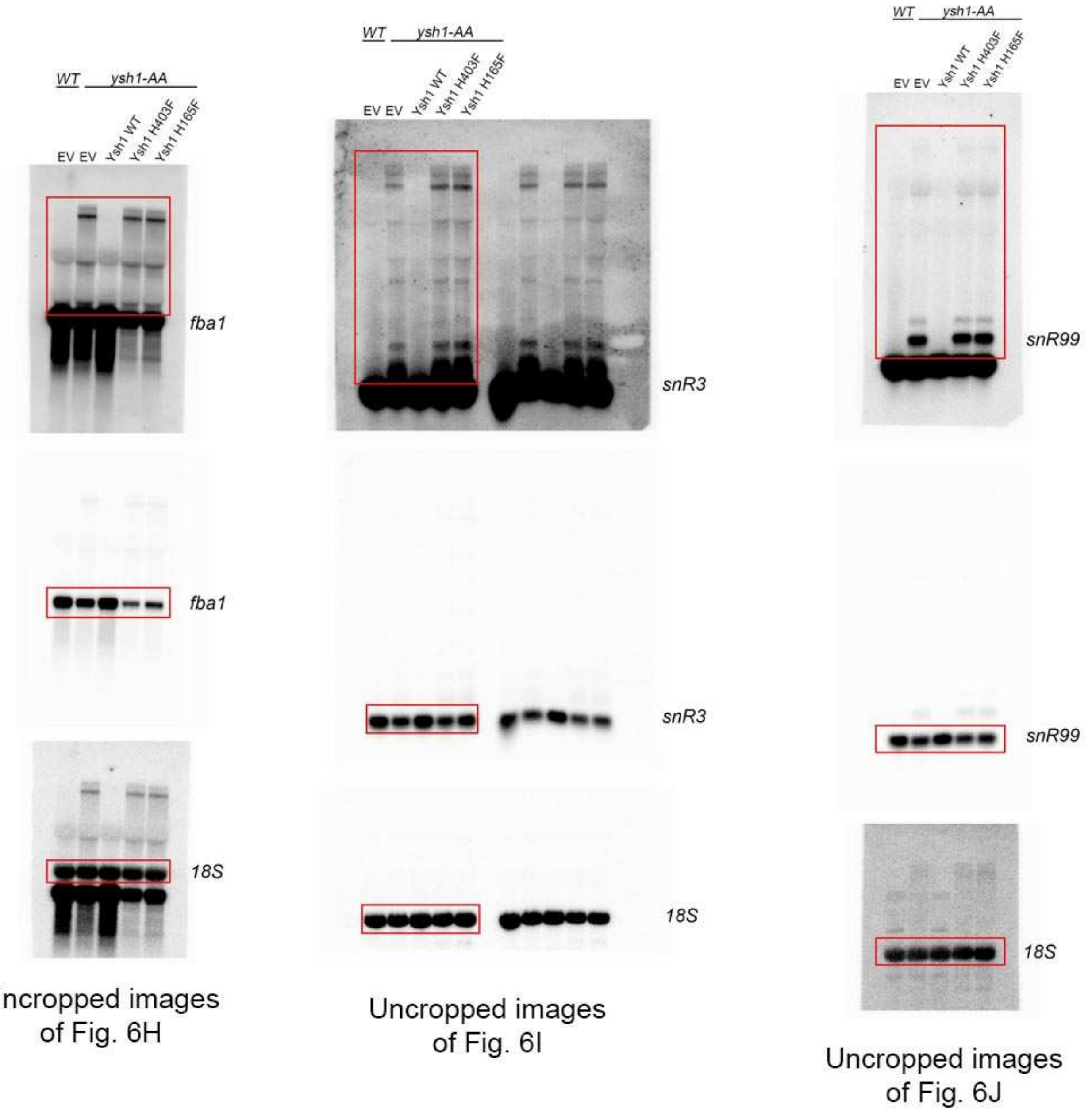

Uncropped images of Fig. 6H

Uncropped images of Fig. 6I

Uncropped images of Fig. 6J

Supplementary Fig. 8. Uncropped raw images from (A) Fig. 2B and Fig. 2C; (B) Fig. 2D, Fig. 2E, and Fig. 2F; (C) Fig. 3F and Fig. 3G; and (D) Fig. 6H, 6I, and 6J.

Supplementary Table 1. List of yeast strains used in this study.

Name	Genotype	Reference
FBY106	h+ ade6-M210 leu1-32 ura4-D18 his3-D1	(3)
FBY107	h- ade6-M216 leu1-32 ura4-D18 his3-D1 pab2::kanMX6	(3)
FBY153	h+ ade6-M210 leu1-32 ura4-D18 his3-D1 Pcf11-2XTAP::kanMX6	(4)
FBY915	h+ ade6-M21? leu1-32 ura4-D18 his3-D1 P41nmt-seb1::kanMX6	(5)
FBY1250	h+ ade6-M216 leu1-32 ura4-D18 his3-D1 snR99::ura4 pFB600::ade6	(5)
FBY1256	h+ ade6-M216 leu1-32 ura4-D18 his3-D1 snR99::ura4 pFB366::ade6	(5)
FBY1275	h+ ade6-M216 leu1-32 ura4-D18 his3-D1 snR99::ura4 pFB622::ade6	This study
FBY1278	h+ ade6-M216 leu1-32 ura4-D18 his3-D1 snR99::ura4 pFB622::ade6 pab2::kanMX6	This study
FBY1357	h+ ade6-M216 leu1-32 ura4-D18 his3-D1 snR99::ura4 pFB652::ade6	This study
FBY1367	h+ ade6-M216 leu1-32 ura4-D18 his3-D1 snR99::ura4 pFB652::ade6 pab2::kanMX6	This study
FBY1409	h? ade6-M21? leu1-32 ura4-D18 his3-D1 P81nmt-pcf11::kanMX6	(6)
FBY1410	h? ade6-M21? leu1-32 ura4-D18 his3-D1 P81nmt-pcf11::kanMX6 pab2::ura4	This study
FBY1496	h? ade6-M21? leu1-32 ura4-D18 his3-D1 nab3::kanMX6	(5)
FBY1507	h? ade6-M21? leu1-32 ura4-D18 his3-D1 sen1::kanMX6	(5)
FBY1516	h? ade6-M21? leu1-32 ura4-D18 his3-D1 dbl8::kanMX6	(5)
FBY1622	h? ade6-M21? leu1-32 ura4-D18 his3-D1 dbl8::kanMX6 sen1::natMX6	(5)
FBY1792	h- ade6-M210 leu1-32 ura4-D18 his3-D1 P81nmt-dhp1::kanMX6	This study
FBY1858	h+ ade6-M216 leu1-32 ura4-D18 his3-D1 Seb1-HTP::kanMX6	(5)
FBY1950	h+ ade6-M216 leu1-32 ura4-D18 his3-D1 Rna14-4XTAP::natMX6	(5)
FBY1956	h+ ade6-M216 leu1-32 ura4-D18 his3-D1 Ysh1-4XTAP::natMX6	(5)
FBY1970	h+ ade6-M216 leu1-32 ura4-D18 his3-D1 Dhp1-4XTAP::ura4	This study
FBY1983	h+ ade6-M216 leu1-32 ura4-D18 his3-D1 Pcf11-4XTAP::natMX6	This study
FBY1985	h? ade6-M21? leu1-32 ura4-D18 his3-D1 kanMX6::P81nmt-Dhp1-4XTAP::ura4	This study
FBY1995	h+ ade6-M216 leu1-32 ura4-D18 his3-D1 Rpb3-3HA::kanMX6	This study
FBY2016	h? ade6-M21? leu1-32 ura4-D18 his3-D1 Rpb3-3HA::kanMX6 P81nmt-dhp1::kanMX6	This study

FBY2066	h- ade6-M210 leu1-32 ura4-D18 torSE::kanMX6 fkh1::URA+ leu1-32::(nmt1-rpl13-2FKBP12-leu1+)	(7)
FBY2109	h- ade6-M210 leu1-32 ura4-D18 torSE::kanMX6 fkh1::URA+ leu1-32::(nmt1-rpl13-2FKBP12-leu1+) Rna14-FRB-GFP::natMX6	This study
FBY2110	h- ade6-M210 leu1-32 ura4-D18 torSE::kanMX6 fkh1::URA+ leu1-32::(nmt1-rpl13-2FKBP12-leu1+) Ysh1-FRB-GFP::natMX6	This study
FBY2161	h+ ade6-M216 leu1-32 ura4-D18 his3-D1 Rpb3-3HA::kanMX6 Pcf11-4XTAP::natMX6	This study
FBY2162	h? ade6-M21? leu1-32 ura4-D18 his3-D1 Rpb3-3HA::kanMX6 Pcf11-4XTAP::natMX6 P81nmt-dhp1::kanMX6	This study
FBY2167	h+ ade6-M216 leu1-32 ura4-D18 his3-D1 Rpb3-3HA::kanMX6 Dis2-4XTAP::natMX6	This study
FBY2170	h? ade6-M21? leu1-32 ura4-D18 his3-D1 Rpb3-3HA::kanMX6 Dis2-4XTAP::natMX6 P81nmt-dhp1::kanMX6	This study
FBY2359	h- ade6-M210 leu1-32 ura4-D18 torSE::kanMX6 fkh1::URA+ leu1-32::(nmt1-rpl13-2FKBP12-leu1+) Ysh1-FRB-GFP::natMX6 pFB1337::ade6	This study
FBY2360	h- ade6-M210 leu1-32 ura4-D18 torSE::kanMX6 fkh1::URA+ leu1-32::(nmt1-rpl13-2FKBP12-leu1+) pFB366::ade6	This study
FBY2361	h- ade6-M210 leu1-32 ura4-D18 torSE::kanMX6 fkh1::URA+ leu1-32::(nmt1-rpl13-2FKBP12-leu1+) Ysh1-FRB-GFP::natMX6 pFB366::ade6	This study
FBY2364	h- ade6-M210 leu1-32 ura4-D18 torSE::kanMX6 fkh1::URA+ leu1-32::(nmt1-rpl13-2FKBP12-leu1+) Ysh1-FRB-GFP::natMX6 pFB1355::ade6	This study
FBY2366	h- ade6-M210 leu1-32 ura4-D18 torSE::kanMX6 fkh1::URA+ leu1-32::(nmt1-rpl13-2FKBP12-leu1+) Ysh1-FRB-GFP::natMX6 pFB1358::ade6	This study
FBY2454	h+ ade6-M216 leu1-32 ura4-D18 his3-D1 natMX6::P41nmt-Seb1-HTP::kanMX6	This study
FBY2455	h+ ade6-M210 leu1-32 ura4-D18 his3-D1 natMX6::P81nmt-Pcf11-2XTAP::kanMX6	This study
FBY2064	MATa ura3-1 leu2-3,112 ade2-1 his3-11,15 trp1-1 can1-100, H2A.Z-3Myc, H2B-3HA (*S. cerevisiae*)	(8)

Supplementary Table 2. List of plasmids used in this study.

Plasmid	Description	Reference
pFB366	Empty ade6 chromosomal integration vector	(9)
pFB600	snR99 noncoding sequence flanked by snR99 promoter and snR99 terminator sequences	(5)
pFB622	snR99 noncoding sequence flanked by snR99 promoter and trx1 terminator sequences	This study
pFB652	snR99 noncoding sequence flanked by snR99 promoter and rps2 terminator sequences	This study
pFB1337	Ysh1-3XFLAG (WT) in pFB366 flanked by ysh1 promoter and terminator sequences	This study
pFB1355	Ysh1-3XFLAG (H165F) in pFB366 flanked by ysh1 promoter and terminator sequences	This study
pFB1358	Ysh1-3XFLAG (H403F) in pFB366 flanked by ysh1 promoter and terminator sequences	This study

Supplementary Table 3. List of primers used in this study.

Name	Sequence	Used for
snR99F1	CCTGAAGTCAAGTATGATGGTTGG	*snR99* ChIP analysis
snR99R1	GGGAAAGCCACTCAATTCTTCGCT	
snR99F2	CGTACCCAAAGTTTGCAATACTACATGA	
snR99R2	TTGAACCCAGCCATCAATGGGA	
snR99F3	GAGCATGATAAAGACGATGTTTGAGG	
snR99R3	CATGAACAGCGATATAGAAAACAAG	
snR99F4	CAGACTTAAGAGTGCGCTAG	
snR99R4	TGCATCCTGGACTACTTCCTC	
snR99F5	AGCAACAGGAGCAGAGGATCAAGT	
snR99R5	TGCAAAGTGCAATCATCCGACACC	
snR3F1	TGACTTAGTTCATCTAGCGACTGC	*snR3* ChIP analysis
snR3R1	GTGACCATAATACCACTTTATATAGCA	
snR3F2	CCCGCATGGTATTTTGCTAT	
snR3R2	CCTTCTGACACTCGCGTACA	
snR3F3	CGCGAAATTCTATATCGCTGT	
snR3R3	AACAGGATTCCAAGCAAACTT	
snR3F4	TGTAATTCTGTGCGATGGGTA	
snR3R4	TCCAAGATGTAGCATGGACAA	
snR3F5	GCAAATTGTGAAGTCAGTATTCGTA	
snR3R5	ATGCATAAAAAGCAGATACCG	
snR3F6	AGCCAACCGAGTGTACCAAG	
snR3R6	CAGGTTATTTCTGTCGTTGCTC	
fba1F1	TCCATCGCTCCTTTCTTCGGTGT	*fba1* ChIP analysis
fba1R1	ACAAGTCAAGCATGTGAGAAGAGA	
fba1F2	GTGAGAAGACCATGACCAAGCGTG	
fba1R2	CAGTATGAACTCTCATTAAGGGGAC	
fba1F3	TGGATTGGATTTGTAAAGTGTTTTTCAAGC	
fba1R3	CATTCATTGTCATGTGGAATCGG	
fba1F4	TCATTGCAATGAGTACGGGCATTTTC	
fba1R4	TTGATGTCTTGACTTGGAGTAAGC	
fba1F5	CATGAAATGCTGCCGACTTTCC	
fba1R5	CGATTTGGAAAGTAATCGTACTTGAACTC	

rps2F1	TCCCAAGGAGCCATTAAAGA	*rps2* ChIP analysis
rps2R1	GGGTAGGGTTCTCTCTCGAA	
rps2F2	AAGTGCGCTAAGGAAGTTGC	
rps2R2	CCCAAAGCAGTACCCCAGTA	
rps2F3	TAACTTTGTTAAGGCTGCCTTTGCC	
rps2R3	CAGCATACTCCTCAATAGGGGTTTG	
rps2F4	GTCACTGTAAGTAAGTTGCACAACA	
rps2R4	CCAATTGATGCACCGGTTAATGCC	
rps2F5	TGAAACCGGCAAGAAGAGA	
rps2R5	TGTTTCCCAATTTTCTTCCTTTT	
rps2F6	TCGACGATAATTTGCCTACTAAA	
rps2R6	TCGTGAAAAGATCACGGAGA	
ncRNA532F1	TTTTCTAAACTTTGCACTCATTTTT	*SPNCRNA.532* ChIP analysis
ncRNA532R1	CATTTCTTCAACCAAATGCAC	
ncRNA532F2	TGAATCCATAATACCCATCTTCAA	
ncRNA532R2	ACCGGGGAGAGAATTTCAAC	
ncRNA532F3	CCAAATACACACCTTTTCTTCCA	
ncRNA532R3	TGCTTTGGGAACAATGACAG	
ncRNA532F4	TCATCAGCCTATGTTCAATTTGT	
ncRNA532R4	TGTCTTGCGCTCATTTCTTG	
ncRNA532F5	AACCTACAGACGAAATACTCTAATGTG	
ncRNA532R5	ATTCATGTATGGATATATTGTTTTCAA	
ncRNA491F1	TCCTGAATCAATTGAAAATCAAC	*SPNCRNA.491* ChIP analysis
ncRNA491R1	CCATCTGGATCCATTATCGTC	
ncRNA491F2	CCCACTTATAGCCTTGGGAAT	
ncRNA491R2	GCTTTGCATAATAAAGCTACCAAA	
ncRNA491F3	TACAAATTGCCCCCACTCG	
ncRNA491R3	CAAATCTGCAGCAATCAACG	
ncRNA491F4	TTTTCTTGCGCGGGATCT	
ncRNA491R4	AAAAACAATGGATGAGAAATTATGG	
ncRNA491F5	GAAGGCTAATTACGGTACCTGCT	
ncRNA491R5	CAAGTTAATTGCAACACACGAAA	
nam1F1	TGGTTTTTGGTTCACGTCAC	*nam1/byr2* ChIP analysis
nam1R1	CATCGAACGCGATGATTTTA	
nam1F2	TTTGGCAGCTTCATTTTGGT	
nam1R2	CTGCCTCCCTTAGTCTCGTG	
nam1F3	CATTGAAAACACCGCCAAAG	
nam1R3	CTCGAGTTTGCCCGTTACAG	
nam1F4	AATTCATCTTCACCGGAACG	
nam1R4	CGGTTGAGCCAATTCAATCT	
nam1F5	AATCCTACCAGCGACCTCCT	
nam1R5	AAGGTTTCTACGAAGAGAGTTTGA	
snR99-NB	CCATCCTTGACCCATGGAAAAAAGTCAACG	Northern blot probe *snR99*
snR3-NB	GTGGACCTTCTGACACTCGCGTACAAGTAGG	Northern blot probe *snR3*
snR99-H	GATGCATTTTCTTGGTCCGGAG	Cleaving oligo RNAseH *snR99*
snR3-H	TCAGTGAATAGCAAATACGAAT	Cleaving oligo RNAseH *snR3*
5S-NB	CCTCAGACGCTTAACTGCAGTGATC	Northern blot

		probe *5S rRNA*
18S-NB	CATGGCTTAATCTTTGAGAC	Northern blot probe *18S rRNA*
fba1-T7	TAATACGACTCACTATAGGGCCTCGGGTTG AGTGTAGAGC	To generate *fba1* T7 riboprobe template
fba1-F	CACGGTGAGCCTCTTTTCTC	
Anchor dT	GGCCACGCGTCGACTAGTACTTTTTTTTTTTTTT TTT	RT 3' RACE
Anchor no dT	GGCCACGCGTCGACTAGTAC	PCR amplification *snR99* 3' RACE
snR99-F	CCTGAAGTCAAGTATGATGGTTGG	

•

SUPPLEMENTARY REFERENCES

1. Liu, X., Hoque, M., Larochelle, M., Lemay, J. F., Yurko, N., Manley, J. L., Bachand, F., and Tian, B. (2017) Comparative analysis of alternative polyadenylation in S. cerevisiae and S. pombe. *Genome Res* **27**, 1685-1695
2. Mata, J. (2013) Genome-wide mapping of polyadenylation sites in fission yeast reveals widespread alternative polyadenylation. *RNA Biol* **10**
3. Perreault, A., Lemieux, C., and Bachand, F. (2007) Regulation of the Nuclear Poly(A)-binding Protein by Arginine Methylation in Fission Yeast. *J Biol Chem* **282**, 7552-7562
4. Lemieux, C., and Bachand, F. (2009) Cotranscriptional recruitment of the nuclear poly(A)-binding protein Pab2 to nascent transcripts and association with translating mRNPs. *Nucleic Acids Res* **37**, 3418-3430
5. Lemay, J. F., Marguerat, S., Larochelle, M., Liu, X., van Nues, R., Hunyadkurti, J., Hoque, M., Tian, B., Granneman, S., Bahler, J., and Bachand, F. (2016) The Nrd1-like protein Seb1 coordinates cotranscriptional 3' end processing and polyadenylation site selection. *Genes Dev* **30**, 1558-1572
6. Larochelle, M., Hunyadkurti, J., and Bachand, F. (2017) Polyadenylation site selection: linking transcription and RNA processing via a conserved carboxy-terminal domain (CTD)-interacting protein. *Curr Genet* **63**, 195-199
7. Ding, L., Laor, D., Weisman, R., and Forsburg, S. L. (2014) Rapid regulation of nuclear proteins by rapamycin-induced translocation in fission yeast. *Yeast* **31**, 253-264
8. Guillemette, B., Bataille, A. R., Gevry, N., Adam, M., Blanchette, M., Robert, F., and Gaudreau, L. (2005) Variant histone H2A.Z is globally localized to the promoters of inactive yeast genes and regulates nucleosome positioning. *PLoS Biol* **3**, e384
9. Beaudoin, J., and Labbe, S. (2006) Copper induces cytoplasmic retention of fission yeast transcription factor cuf1. *Eukaryot Cell* **5**, 277-292
