# Supplementary material for: Common mechanism of transcription termination at coding and noncoding RNA genes in fission yeast
Source: Nat Commun. 2018 Oct 19;9:4364. doi: 10.1038/s41467-018-06546-x (PMC6195540; doi:10.1038/s41467-018-06546-x)
Supplement: Supplementary file 3 — Description of Additional Supplementary Files [file 41467_2018_6546_MOESM3_ESM.pdf]

Description of Additional Supplementary Files:

Supplementary Data 1. List of mRNA genes used in this study in genePred format, including those modified according to the strongest polyadenylation site as determined by reanalyzis of 3'READS data.

Supplementary Data 2. List of monocistronic snoRNA genes used in this study in genePred format, including those modified according to the strongest polyadenylation site as determined by reanalyzis of 3'READS data.

Supplementary Data 3. Summary of sequencing statistics resulting from the ChIP-seq data.
